# Consumer acceptance of autonomous delivery robots for last-mile delivery: Technological and health perspectives

**DOI:** 10.3389/fpsyg.2022.953370

**Published:** 2022-09-09

**Authors:** Kum Fai Yuen, Lanhui Cai, Yong Guang Lim, Xueqin Wang

**Affiliations:** ^1^School of Civil and Environmental Engineering, Nanyang Technological University, Singapore, Singapore; ^2^Department of International Logistics, Chung-Ang University, Seoul, South Korea

**Keywords:** autonomous delivery robots, COVID-19, technology acceptance model, health belief model, perceived value, perceived trust

## Abstract

The unprecedented outbreak of the novel coronavirus has led to a great shift toward online retailing and accelerated the need for contactless delivery. This study investigates how technological and health belief factors influence consumer acceptance of autonomous delivery robots (ADRs). Anchored in four behavioral theories [i.e., technology acceptance model, health belief model, perceived value (VAL) theory and trust theory], a synthesized model is developed. A total of 500 valid responses were collected through an online questionnaire in Singapore, and structural equation modeling was conducted to examine the responses. The results revealed that perceived ease of use (EOU), perceived usefulness (UFN), perceived susceptibility (SUS), perceived severity (SEV), self-efficacy (SEL) and cues to action (CUE) have a positive and significant influence on consumers’ perceptions of the value of ADRs. The total effect analysis also showed that perceived VAL strongly affects consumer acceptance of ADRs. Academically, this study introduces both technological and health belief factors to explain consumer acceptance of ADRs. It also provides recommendations for policymakers and autonomous delivery robot developers on policy formulation, public communication, product design and infrastructure development.

## Introduction

Last-mile delivery is the final stage of the business-to-consumer delivery process that involves delivering goods to the end customer (Yuen et al., 2019). The accelerated growth of e-commerce stimulated by online retailing has led logistics companies to develop sustainable and affordable last-mile delivery innovations, such as automated parcel lockers, low-altitude drones and delivery robots. These innovations can address considerable issues, such as traffic congestion, misdeliveries and workforce shortages. With the unprecedented outbreak of the novel coronavirus (COVID-19) pandemic since the beginning of 2020, e-commerce has further expanded as more consumers have shifted toward online retailing (Park and Lee, 2021). COVID-19 has since accelerated the need for contactless delivery and changed the societal needs of consumers.

Capable of facilitating contactless delivery, autonomous delivery robots (ADRs) are increasingly being used to support last-mile delivery during the ongoing COVID-19 pandemic. The use of ADRs can protect both the consumers and the delivery drivers against coronavirus by minimizing potential exposure (Pani et al., 2020). Additionally, ADRs can provide flexibility and convenience for consumers, as they can allow consumers to select their preferred delivery time (Chen C. et al., 2021). ADRs can also ensure safe and secure deliveries, as consumers can only retrieve their goods from the ADRs after verifying their identities by scanning a quick response (QR) code or entering a one-time password.

Despite the abovementioned benefits of using ADRs, challenges also impede consumers’ adoption and acceptance of ADRs. First, compared to traditional home deliveries, which require no consumer participation, consumers may be reluctant to accept the use of ADRs, as they may perceive that it is less convenient. Second, consumers who are less technologically inclined may experience technical difficulties with ADRs (Collier et al., 2015). There may also be a lack of trust among consumers in the reliability and effectiveness of ADRs, as it is a newly emerged delivery innovation. Moreover, considering that ADRs involve data privacy and confidentiality issues, the perceived risk is also a factor in consumer acceptance (Alonso et al., 2021; Leon et al., 2021). Apart from these challenges from the consumer perspective, operational challenges such as the need for a barrier-free infrastructure and theft prevention mechanisms may also affect the acceptance of ADRs.

While companies globally have already started to adopt ADRs to perform last-mile delivery, Singapore has begun to conduct trials to deliver food and parcels with ADRs as part of the nation’s efforts to become a Smart Nation. However, due to the emergence of new COVID-19 variants, which are more lethal and transmissible, such as the Alpha, Delta and Omicron variants, Singapore has further accelerated its digitalization program (IMDA, 2020). As a result, more ADR trials are being conducted. For instance, ADR trials are being conducted at Punggol Waterway Woodcress, a residential estate in northern Singapore, to deliver groceries bought from supermarkets and parcels containing goods ordered online (Tham, 2021). ADR food delivery trials are being conducted between eateries and a commercial building at Jurong East in western Singapore (Abdullah, 2021). These trials are being conducted in tandem while Singapore embarks on its journey toward becoming a COVID-19 Resilient Nation.

As mentioned previously, existing challenges can impede consumer acceptance of ADRs. Thus, there is an urgent need to identify the factors that impact the behavioral intention and consumer acceptance of ADRs. To explain these factors, past theoretical research has been conducted, and these theories include the extended unified theory of acceptance and use of technology (Kapser and Abdelrahman, 2020), diffusion of innovation theory (Wang et al., 2020), technology acceptance model and theory of reasoned action (Felch et al., 2019). A major focus of these theories is the influence of technical characteristics on consumer acceptance of ADRs.

However, with the outbreak of the COVID-19 pandemic, the abovementioned theories are no longer sufficient, as external environmental factors have changed due to the pandemic. In particular, other environmental factors, such as health belief concerns, may also influence consumer acceptance. Research has suggested that the COVID-19 pandemic has created a shift toward online retailing and accelerated consumers’ need for contactless delivery. Few studies on acceptance of ADRs address the pandemic’s external environment and health factors. Thus, it is evident that a research gap exists whereby there is a lack of investigation on how other environmental factors will impact consumer acceptance of technology in this COVID-19 landscape, in particular ADRs, as they are a relatively new last-mile delivery innovation.

In view of the research gap, this study investigates how both the technological and health belief factors will influence consumer acceptance of ADRs by building on a theoretical model anchored on two theories: (1) The technology acceptance model (Yaprak et al., 2021), which explains the acceptability of technology with perceived ease of use (EOU) and perceived usefulness (UFN), and (2) the health belief model (Yuen et al., 2020a), which explains how the health belief concerns influence consumers’ trust and consequently its acceptance. The health belief model proposes that individuals’ decisions to engage in health self-protection behaviors are influenced by perceived threat, outcome expectation, cues to action (CUE) and self-efficacy (SEL). Adopting ADRs can be viewed as a self-protection behavior, as it enables individuals to protect themselves against a virus during face-to-face interactions throughout a pandemic.

This study further differentiates itself from other studies by introducing two additional theories as mediators into the model. These mediating theories include (1) perceived value (VAL) theory, which explains the concept of utility behind consumers’ decisions, and (2) trust theory, which explains how consumers’ trust in technology may lead to consumer acceptance (Yuen et al., 2021a). Accordingly, this multi-dimensional model is integrated into the stimulus-organism-response (SOR) framework, which acts as an overarching structure (Li et al., 2021b). The remainder of this paper is structured as follows. Firstly, a model consisting of a network of research hypotheses based on the aforementioned theories is developed to justify consumer acceptance of ADRs. Secondly, the research methods used for data collection and model analysis are presented. Thirdly, the results and analyses are discussed. Finally, the theoretical contributions and practical implications are explained, and future research directions are recommended.

## Literature review

### Stimulus-organism-response framework

The three-stage SOR framework, which acts as an overarching structure in the theoretical model of this study, has been widely used to investigate how human behavior changes in response to external stimuli (Li et al., 2021b). The three stages in the SOR framework include stimulus, organism and response. The stimulus refers to the external stimuli that trigger the cognitive and affective mechanisms, which can affect the attitudes and decision-making of individuals (Lavuri, 2021). The organism, which acts as a mediator between stimulus and response, refers to individuals’ internal processes based on cognitive and affective reactions to external stimuli (Islam et al., 2021). The response refers to the changes in behavior, intention and decision-making in response to the “stimulus” and “organism” factors (Sung et al., 2021).

As the SOR framework provides a basic logical framework for studying the influence of various stimuli on consumer behavior, it has been used in a broad field of research in the context of the COVID-19 pandemic. This study aims to explore consumer acceptance of ADRs in the context of the pandemic. To this end, the SOR framework serves as the basic framework.

### Stimulus (technology acceptance model and health belief model)

The technology acceptance model has been used extensively to study the behavioral intentions and consumer acceptance of new technological innovations (Alaimo et al., 2020; Sung and Jeon, 2020). The technology acceptance model includes perceived EOU and perceived UFN, highlighting the impact of technical characteristics on consumer acceptance. As this study investigates a relatively new last-mile delivery innovation, the technology acceptance model developed by Davis (1989) is applied. The two factors of the technology acceptance model are applied to the SOR framework as the technological stimuli.

Consumer acceptance of ADRs has previously been studied in terms of technology-related factors. The study of Kapser and Abdelrahman (2020) used UTAUT to consider consumer acceptance of ADRs, and Pani et al. (2020) considered locational, psychological and psychological factors affecting consumer acceptance of ADRs. However, these studies ignored health-related factors, which may affect consumer acceptance of ADRs in the context of the COVID-19 pandemic. As this study was conducted during the COVID-19 pandemic, it focused on the health belief concerns of consumers, which are increasingly important when investigating consumer acceptance of new technological innovations. The factors in the health belief model include perceived benefits, perceived barriers, perceived susceptibility (SUS), perceived severity (SEV), SEL and CUE. Thus, the health belief model developed by Hochbaum et al. (1952) explains the health protection behavior of individuals and is also applied to this study. However, since both perceived benefits and perceived barriers are synonyms and antonyms of the perceived UFN of the technology acceptance model, respectively, they are omitted from the theoretical model of this study. As a result, the remaining factors of the health belief model are applied to the SOR framework as the environmental stimuli.

Considering that the ongoing COVID-19 pandemic only started in early 2020, previous studies did not investigate the health belief concerns of consumers, which could have a significant influence on the perceived VAL of ADRs. Therefore, specific constructs in the technology acceptance model and the health belief model are proposed to provide an all-encompassing view to better understand consumers’ creation of perceived VAL of ADRs. Therefore, the constructs including perceived EOU, perceived UFN, perceived SUS, perceived SEV, SEL and CUE are considered as stimulating factors in this study.

### Organism (perceived value theory and perceived trust theory)

In this study, the perceived VAL theory is applied to operationalize the cognitive and affective mechanisms. According to Yuen et al. (2019), perceived VAL consists of four dimensions (i.e., functional, economic, social, and hedonic). It is the utility derived after assessing the benefits and costs of purchasing a product and/or service. In other words, when individuals evaluate that the benefits outweigh the costs, positive perceived VAL is achieved. Several factors that influence consumers’ perceived VAL for the use of technology have been recognized by previous studies. For instance, Yuen et al. (2019) revealed that convenience, reliability and privacy security, which are resource matching characteristics, have a significant influence on consumers’ perceptions of the value of using smart lockers, which is a form of last-mile delivery innovation. Additionally, attributes such as effort expectancy, performance expectancy and perceived risk also have a notable influence on individuals’ perceptions of the value of using information technology platforms for financial services (Xie et al., 2021). However, with ADRs being a relatively new phenomenon for last-mile delivery, there are currently few studies on the determinants of consumers’ perceptions of the value of ADRs.

According to Castelfranchi and Falcone (2010), perceived trust (TRU) is the belief that a trustee with specific attributes will provide positive results for the trustor by performing certain actions. In other words, positively perceived TRU is created when the benefits outweigh the associated costs (Yuen et al., 2020a). The trust theory is also applied in this study. In short, both the perceived VAL theory and trust theory are applied in the SOR framework of this study as “organisms” to investigate how perceived VAL and perceived TRU influence consumer acceptance of ADRs.

## Theoretical model and hypothesis development

**FIGURE 1:**
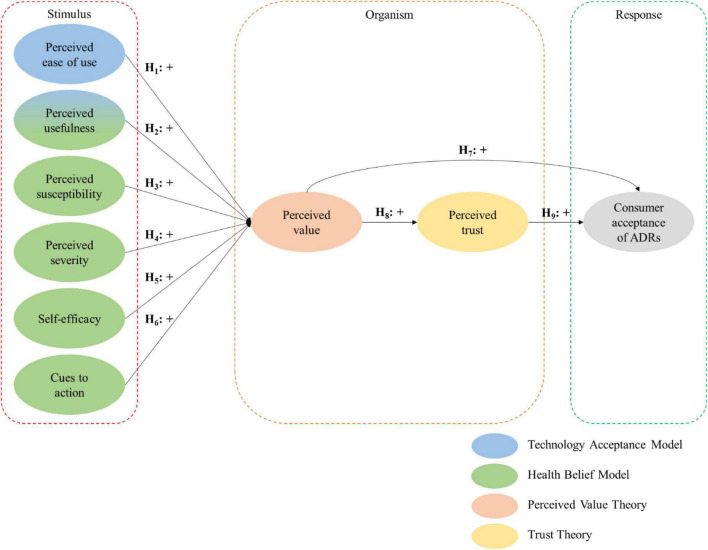
Theoretical model.

As depicted in Figure 1, this study synthesizes four behavioral theories integrated into the SOR framework to investigate consumer acceptance of ADRs as a last-mile delivery innovation. The four theories are the (1) technology acceptance model, (2) health belief model, (3) perceived VAL theory and (4) trust theory. Table 1 illustrates the basic assumptions, the constructs representing each theory and each theory’s contributions to the theoretical model.

**TABLE 1:** Appraisal of behavioral theories that influence consumer acceptance of ADRs.

Perspective	Innovation acceptance	Psychology	Consumer utility	Psychology
Basic assumptions	Consumer acceptance of an innovation is affected by its technological characteristics.	Consumer acceptance of an innovation is influenced by health belief concerns and its characteristics.	Products and/or services that maximize consumers’ utility will be chosen and adopted.	The positive expectation about the performance of the innovation can lead to consumer acceptance, in circumstances where perceived risk is consistent.
Representative construct(s)	Perceived ease of use and perceived usefulness	Perceived usefulness, perceived susceptibility, perceived severity, self-efficacy, and cues to action	Perceived value	Perceived trust
Theory’s contributions to model	The formation of perceived value can be justified by using the two technology acceptance characteristics via this theory.	The creation of perceived value can be justified by using the various health belief characteristics via this theory.	Using the technological and environmental (i.e., health belief) stimuli, this theory can justify the building of perceived value, which ultimately leads to consumer acceptance of ADRs.	The influence on perceived trust of consumers toward the use of ADRs can be justified via this theory with consistent perceived value, which eventually leads to their acceptance.

### Theoretical model

To explain the hypotheses as presented in Figure 1, three key arguments are presented in this study. The first argument relates to the antecedents of perceived VAL. Since the use of ADRs is considered as a form of innovation to cater to new societal needs in the COVID-19 landscape, the technology acceptance model and the health belief model can be used to determine the characteristics of ADRs leading to positive perceived VAL. In this study, the technological and health belief characteristics are used to justify the creation of functional, economic, social and hedonic utility, which increases the perceived VAL of ADRs among consumers (H_1_–H_6_).

The second argument stems from the perceived VAL theory. When a product or service maximizes utility among the alternative offerings in the market, it can foster consumer acceptance, which justifies the positive relationship between perceived VAL and consumer acceptance of ADRs. In other words, if the ADRs can provide superior value to the consumers compared with other last-mile delivery innovations, it can promote the acceptance of ADRs (H_7_).

The final argument is related to the indirect effect of perceived VAL on consumer acceptance of ADRs through perceived TRU. In particular, when using ADRs produces positive utility for consumers, perceived TRU is formed, as its benefits outweigh its associated costs and perceived risks (H_8_). Further, when consumers possess high levels of perceived TRU, it can enhance their confidence, consequently motivating and leading to their acceptance of ADRs (H_9_).

### Hypothesis development

#### Influence of perceived ease of use on consumers’ perceptions of value of autonomous delivery robots

This study refers to the perceived EOU as the level of complexity of using ADRs for last-mile delivery (Yuen et al., 2021b). Generally, if the use of ADRs is simple or less complex, consumers will require little time and resources to learn how to use them. As a result, when compared to learning other more complex technologies, there will be time and resource savings that can be otherwise utilized. Consequently, the economic utility will be enhanced, which increases the perceived VAL of ADRs. Therefore, a positive influence of perceived EOU on consumers’ perceptions of the value of ADRs is proposed.

***H***_1_: *Perceived ease of use has a positive influence on consumers’ perceptions of the value of ADRs.*

#### Influence of perceived usefulness on consumers’ perceptions of value of autonomous delivery robots

Perceived UFN refers to the benefits that ADRs can bring to consumers and the ability to meet consumers’ personal needs (Felch et al., 2019). The use of ADRs can provide convenience, as the preferred delivery time can be chosen by consumers. Although it is argued that other delivery methods such as home deliveries can also allow consumers to select their preferred delivery time, home deliveries tend to have longer delivery time windows, which create uncertainty for consumers (Köhler et al., 2020). With the use of ADRs, consumers can experience higher certainty in delivery time, and waiting through long delivery time windows is not required. Moreover, as contactless delivery has become a new societal need in the pandemic, the ability to conduct contactless delivery has become increasingly important. Since ADRs operate autonomously without any human intervention, contactless delivery is possible. As a result, using ADRs will enhance both the functional and social utility for consumers.

Additionally, as the use of ADRs is considered a new last-mile delivery innovation, the experience that consumers acquire from using ADRs can also satisfy the potential desire that consumers may have to try something new, consequently enhancing the hedonic utility for consumers. Therefore, a positive impact of perceived UFN on consumers’ perceptions of the value of ADRs is proposed.

***H***_2_: *Perceived UFN has a positive influence on consumers’ perceptions of the value of ADRs.*

#### Influence of perceived susceptibility on consumers’ perceptions of value of autonomous delivery robots

In this study, perceived SUS relates to the consumers’ subjective evaluation of their risks of contracting COVID-19 (Yuen et al., 2021c). Medical studies have been conducted recently to understand the vaccine efficacy against COVID-19, and results have revealed that vaccine efficacy is reduced with the emergence of new variants such as the Alpha, Delta and Omicron variants (Bernal et al., 2021). This is also evident in Singapore, where even with more than 85% of the Singapore population are fully vaccinated, there is still a surge in COVID-19-positive cases (McGregor, 2021).

The use of ADRs can offer contactless delivery whereby consumers do not have to be in contact with delivery drivers, and this can minimize consumers’ potential exposure to COVID-19. Additionally, ADRs allow consumers to get their online purchases delivered, which can reduce their need to make purchases at physical retail stores, further reducing the likelihood of getting quarantine orders and stay-home notices. On the whole, the use of ADRs can reduce consumers’ risk of contracting COVID-19.

Being able to facilitate contactless delivery and deliver online purchases, ADRs can provide functional utility to consumers. Furthermore, the reduction in the likelihood of getting quarantine orders and stay-home notices can provide economic utility to consumers, as a loss of income from work absenteeism for working adults can be prevented (Bodas and Peleg, 2020). Thus, a positive impact of perceived SUS on consumers’ perceptions of the value of ADRs is proposed.

***H***_3_: *Perceived susceptibility has a positive influence on consumers’ perceptions of the value of ADRs.*

#### Influence of perceived severity on consumers’ perceptions of value of autonomous delivery robots

Perceived SEV is the consumers’ assessment of the seriousness of the adverse consequences of contracting COVID-19 (Yuen et al., 2020a). These negative consequences can include death after succumbing to COVID-19 and hospitalization due to the need for oxygen supplementation and intensive care (Hussein et al., 2021). Additionally, adverse consequences also include the repercussions after contracting COVID-19, such as loss of income from work absenteeism and mental health problems that arise from prolonged isolation and stress in complying with public health regulations (Giorgi et al., 2020).

With the likelihood of contracting COVID-19 being reduced after the use of ADRs, the seriousness of adverse consequences that consumers may suffer will also be reduced as a result. In particular, the prevention of loss of income and mental health problems can improve consumers’ economic and hedonic utility. Hence, a positive impact of perceived SEV on consumers’ perceptions of the value of ADRs is proposed.

***H***_4_: *Perceived severity has a positive influence on consumers’ perceptions of the value of ADRs.*

#### Influence of self-efficacy on consumers’ perceptions of value of autonomous delivery robots

SEL is consumers’ level of confidence in their ability to facilitate decisions and allocate resources to learn and use ADRs (Chen H. et al., 2021). To enhance consumers’ confidence level in using ADRs, various ADR trials have been conducted in different parts of Singapore. Furthermore, support resources such as government campaigns and advertisements have been launched to create awareness among consumers. With the allocation of these support resources, consumers can gain confidence in their ability to learn and use ADRs.

The use of ADRs will not only fulfill the consumers’ needs by conducting contactless delivery for their online purchases but also allow consumers who are aware of this last-mile delivery innovation to be confident enough to recommend it to their family and friends. This will in turn enhance both the functional and social utility for consumers. Therefore, a positive influence of SEL on consumers’ perceptions of the value of ADRs is proposed.

***H***_5_: *Self-efficacy has a positive influence on consumers’ perceptions of the value of ADRs.*

#### Influence of cues to action on consumers’ perceptions of value of autonomous delivery robots

CUE refer to internal and external factors facilitating the intention to use ADRs (Johnson et al., 2021). Specifically, internal factors can include the personal experiences of consumers who have participated in the ADR trials conducted, whereas external factors can include word-of-mouth communication by family and friends and awareness programs such as government campaigns and advertisements.

According to Jafarzadeh et al. (2021), who employed the cognitive dissonance theory, consumers with negative experiences may develop negative attitudes, while consumers with positive experiences are likely to use the product or service again. This signifies that if consumers personally have positive experiences, or have heard recommendations from family and friends on the use of ADRs, they are likely to choose ADRs as their preferred last-mile delivery method. Additionally, as the use of ADRs is still relatively new, consumers who are aware of this technology but have yet to experience it may be excited to give it a try. As such, this can enhance both the functional and hedonic utility for consumers. Thus, a positive impact of CUE on consumers’ perceptions of the value of ADRs is proposed.

***H***_6_: *Cues to action have a positive influence on consumers’ perceptions of the value of ADRs.*

#### Direct influence of perceived value on consumers’ acceptance of autonomous delivery robots

This study suggests that perceived VAL directly influences consumers’ acceptance of ADRs for last-mile delivery, and the perceived VAL theory can justify their acceptance of ADRs. In particular, the theory posits that if consumers perceive that the four value dimensions (i.e., functional, economic, social and hedonic utility) are met, they would be more motivated to use ADRs, as they wish to experience the benefits and utilities that can be offered in reality (Yuen et al., 2020b).

Additionally, if consumers perceive that the use of ADRs offers higher utility levels compared to other last-mile delivery methods, they are more likely to accept and commit to the use of ADRs. Therefore, a direct and positive influence of perceived VAL on consumers’ acceptance of ADRs is proposed.

***H***_7_: *Perceived value has a positive influence on consumers’ acceptance of ADRs.*

#### Indirect influence of perceived value on consumers’ acceptance of autonomous delivery robots via perceived trust

This study also suggests that perceived VAL indirectly influences consumers’ acceptance of ADRs via perceived TRU. According to Castelfranchi and Falcone (2010), if there is an increase and decrease in the expected benefits and associated costs, respectively, trust can be enhanced.

In this study, the increased perceived VAL using ADRs can increase the expected benefits. For instance, an increase in perceived EOU increases the benefits for consumers in terms of economic utility, as they can enjoy time and resource savings when learning and using ADRs. These benefits and utilities can create incentives for consumers to trust ADRs.

Not only that, the increase in perceived VAL using ADRs can also decrease the associated costs. For example, using ADRs can reduce the risks of contracting COVID-19, as they can conduct contactless delivery. This reduction in related costs can also create incentives for consumers to trust ADRs. With both the increase in the expected benefits and the decrease in associated costs, a positive influence of perceived VAL on perceived TRU in ADRs is proposed.

***H***_8_: *Perceived value has a positive influence on consumers’ perceived trust in ADRs.*

The trust that consumers have in ADRs is important for their acceptance and adoption, and it can be increased when the ADRs exhibit delivery expertise, reliability, integrity and competency (Lee and Kolodge, 2020; Yuen et al., 2020b). As ADRs are fully autonomous and operated without any human intervention, a high level of trust is required from consumers, as they possess minimal control over the execution of delivery tasks. If the ADRs can perform the delivery tasks accurately and promptly, high levels of trust can be generated. This can consequently enhance consumers’ confidence in ADRs, resulting in their acceptance. Therefore, a positive impact of perceived TRU on consumers’ acceptance of ADRs is proposed.

***H***_9_: *Perceived trust has a positive influence on consumers’ acceptance of ADRs*.

## Methodology

Structural equation modeling (SEM) is employed to test the theoretical model and hypotheses, as it is capable of analyzing the unobservable and multifaceted relationships between the latent variables (Streiner, 2006). Further, this method is chosen, as it can improve the model estimation accuracy and approximate the parameters of the model simultaneously. Finally, SEM provides a variety of fit indices to evaluate the fitness of the proposed model and data.

### Measurement items

Modified from past studies, measurement items are introduced to measure each construct. These constructs include six stimuli [perceived EOU, perceived UFN, perceived SUS, perceived SEV, SEL, CUE], two organism factors [perceived VAL, perceived TRU], and one response factor [consumer acceptance of ADRs (ACC)]. Table 2 summarizes the measurement items and their corresponding constructs, the central themes and the supporting literature.

**TABLE 2:** Modified measurement items and their corresponding constructs.

Construct	ID	Measurement items	Central themes	Supporting literature
Perceived ease of use (EOU)		*Strongly disagree (1)/Strongly agree (7)*		
	EOU1 EOU2 EOU3 EOU4 EOU5	I believe that learning how to use ADRs would be easy. I believe that the use of ADRs would be clear and easy to understand. I believe that it is easy for me to become skillful at using ADRs. I believe that it is easy for me to get ADRs to do what I want them to do. I believe that interacting with ADRs would not require much of my mental effort.		Manis and Choi, 2019; Yuen et al., 2021b
**Perceived usefulness (UFN)**				
	UFN1 UFN2 UFN3 UFN4 UFN5	ADRs will be useful to me. ADRs can help me to live a normal life. ADRs can help me to improve my efficiency. ADRs would increase my flexibility in my daily life. ADRs can satisfy my desire to try something new.	Functional benefit Social benefit Economic benefit Economic benefit Hedonic benefit	Zhang et al., 2019; Kapser and Abdelrahman, 2020; Li et al., 2021a
**Perceived susceptibility (SUS)**				
	SUS1SUS2SUS3 SUS4‘	I am more likely to contract COVID-19 because of my physical health. The likelihood of me contracting COVID-19 in the future is high. I worry a lot about contracting COVID-19. I am more likely to contract COVID-19 if I do not use ADR services.	Susceptibility (Self) Susceptibility (Self) Susceptibility (Self) Susceptibility (External)	Huang et al., 2016; Yuen et al., 2021a
**Perceived severity (SEV)**				
	SEV1 SEV2 SEV3 SEV4	COVID-19 would threaten my health to a great extent. The thought of contracting COVID-19 terrifies me. COVID-19 would threaten my physical and mental health to a great extent. I fear any long-term economic losses/effects after contracting COVID-19.	Severity (Physical) Severity (Physical) Severity (Physical/Hedonic) Severity (Economic)	Huang et al., 2016; Li et al., 2021a;Yuen et al., 2021a
**Self-efficacy (SEL)**				
	SEL1 SEL2 SEL3 SEL4 SEL5	I would have the resources necessary to use ADRs. I would have the knowledge necessary to use ADRs. I would be able to get help from others if I had difficulties using ADRs. ADRs would be compatible with my current lifestyle and habits. I have the ability to learn how to use ADRs easily.		Venkatesh et al., 2012; Li et al., 2021a
**Cues to action (CUE)**				
	CUE1 CUE2 CUE3 CUE4	My personal experience with ADRs would prompt me to use them again. My family and friends would support me if I used ADRs. I am highly encouraged by the government to use ADRs. I will only use ADRs if more people repeatedly use them.	Internal cues External cues External cues External cues	Huang et al., 2016; Yuen et al., 2021a
**Perceived value (VAL)**				
	VAL1 VAL2 VAL3 VAL4	I feel that ADR services will be reasonably priced as compared to other delivery methods. I feel that using ADRs to deliver my purchases will be effective and efficient. I feel that using ADRs will be pleasant. I feel that using ADRs would have positive effects on the environment and society.	Economic value Functional value Hedonic value Social value	Yuen et al., 2019
**Perceived trust (TRU)**				
	TRU1 TRU2 TRU3 TRU4	I trust that ADRs can perform deliveries without assistance from me. I trust that ADRs are safe and reliable in severe weather conditions. I would trust ADRs more than human-intervened delivery options. ADRs can be trusted to carry out deliveries effectively.	Expertise Reliability Integrity Competency	Kaur and Rampersad, 2018; Nordhoff et al., 2018
Consumer acceptance of ADRs (ACC)		*Extremely unlikely (1)/Extremely likely (7)*		
	ACC1 ACC2 ACC3 ACC4	I intend to use ADRs to deliver my purchases in the future. I would consider the use of ADRs to be the first choice for my purchases. I would recommend the use of ADRs to my family and friends. I would say positive things about ADRs to my family and friends.		Choi and Ji, 2015; Zhao et al., 2018

### Design and administration of questionnaire

The measurement items introduced were integrated into an online questionnaire for data collection, and the questionnaire had three segments. The first segment was the introduction of the questionnaire, which included the study’s objective and some background information about ADRs and its trials conducted in Singapore. This section also contained a statement that assured the survey respondents that their identities and responses were anonymous, in an attempt to encourage honest responses. The second segment asked about the respondents’ demographics, like gender, age, employment status, monthly household income, highest attained education level and housing type. Additionally, there were questions on the respondents’ vehicle ownership, preferred mode of delivery, vaccination status and history of contracting COVID-19. Finally, the third segment presented the respondents with the measurement items in Table 2, and they were required to provide a rating on a 7-point Likert scale based on their level of agreeableness and the likelihood of occurrence (Likert, 1932).

The questionnaire was firstly designed, and a professional market research company, Rakuten Insights, was appointed to administer the questionnaire put onto the Qualtrics survey platform on a panel of respondents. Several partnering panels were blended to create a representative sampling frame. Initially, the questionnaire was designed and made partly available to *n* = 50 respondents, and some modifications were then made based on the responses gathered to improve the clarity of the measurement items. The refined questionnaire was officially launched for data collection of respondents living in Singapore. During the data collection process, respondents were required to pass the qualifying tests, which included two attention checkers (i.e., choosing a specific answer) that were mixed in with the formal questions to ensure the responses’ reliability. Respondents who failed the qualifying tests were disqualified, as they did not pay attention while responding to the questionnaire. Finally, of the 1,543 online survey questionnaires distributed by Rakuten Insights, 500 valid responses were collected for analysis. The effective response rate was 32.4%.

### Respondents’ demographics

Table 3 presents the demographics of the 500 respondents who successfully completed the questionnaire. Of the respondents, 49 and 51% were males and females, respectively, which reflect representativeness, as the gender distribution of the Singapore population is 50% males and 50% females. This is in accordance with the Singapore Census of Population 2020 (DOS, 2021). Next, about 40% of the respondents were above 40 years old, and this was almost consistent with the population’s median age of 41.8 years reported in 2021 (DOS, 2022). Furthermore, 30% of the respondents reported a monthly household income of $10,000 and above, which was relatively close to the 40% reported in the national income statistics.

**TABLE 3:** Demographic profile of respondents.

	Frequency	Proportion (%)
**Gender**		
Male	246	49
Female	254	51
Age*		
16–34 years	220	44
35–49 years	190	38
≥50 years	90	18
**Employment status**		
Employed	387	77
Self-employed	35	7
Unemployed	28	6
Student	30	6
Other	20	4
Monthly household income (SGD)*		
≤2,999	92	18
3,000–9,999	258	52
10,000–14,999	108	22
≥15,000	42	8
Highest attained education level*		
Primary and below	3	1
Secondary	54	11
Junior college	34	7
Polytechnic	129	26
Undergraduate	209	42
Postgraduate	71	14
**Housing type**		
Public housing (HDB Flat)	414	83
Condominium	75	15
Landed	11	2
**Ownership of vehicle**		
Yes	209	42
No	291	58
**Preferred mode of delivery**		
Home delivery	442	88
Self-collection	58	12
**Vaccination status**		
Fully vaccinated (at least two doses of mRNA or three doses of Sinovac vaccines)	489	98
Partially or not vaccinated (less than two doses of mRNA or three doses of Sinovac vaccines)	11	2
**History of Contracting COVID-19**		
Yes	61	12
No	439	88

*Control variable used in the theoretical model.

HDB denotes housing and development board, Singapore.

Additionally, 83% of the respondents resided in public housing, and this was similar to the national average of 80% (HDB, 2020). Corresponding to Singapore’s focus on providing quality education, 89% of the respondents received more than secondary education. Finally, Rakuten Insights found that 98% of the respondents had been fully vaccinated, and this figure was almost consistent with the 91% vaccination rate as of March 2022 (MOH, 2022). Based on the above comparisons, the sample was representative.

### Bias examination

As self-administrated questionnaires were applied in this study whereby both the independent and dependent variables were derived from the same group of respondents, two possible bias phenomena could have compromised the validity of the survey results. Firstly, non-response bias was examined, where late respondents may present with specific characteristics similar to non-respondents. The 500 responses collected were split equally into two groups (i.e., 250 in each group) according to the completion time (Armstrong and Overton, 1977). Following this, an independent *t*-test was performed, and the test results revealed a non-significant difference (i.e., *p* > 0.05). Thus, no non-response bias existed in this study.

Secondly, as the survey responses were collected via a single medium (i.e., online means), common method bias was possible, where survey responses may be skewed or show a certain tendency that could understate or overstate the results. Hence, Harman’s single factor test was performed where all items were specified to load on a single factor to determine the level of common method bias. The total variance of the test showed that it was below the recommended critical value of 50%; thus, no common method bias existed in this study (Podsakoff et al., 2003).

## Results and discussion

### Analysis of measurement model

Confirmatory factor analysis (CFA) was performed to examine the fitness of the measurement model as a whole and to analyze the reliability and validity of the measurement items. Accordingly, the results of the CFA are presented in Table 4. The fit indices of the measurement model are also displayed in Table 4, and these fit indices include χ^2^*/df* = 2.106 (*p* < 0.05), CFI = 0.96, TLI = 0.97, RMSEA = 0.058 and SRMR = 0.062. These fit indices passed the recommended threshold proposed by Hu and Bentler (1999), thus suggesting that the measurement model possessed good overall fitness.

**TABLE 4:** Results of CFA.

Construct	Item	λ	AVE	CR
Perceived ease of use (EOU)	EOU1 EOU2 EOU3 EOU4 EOU5	0.839 0.886 0.901 0.878 0.839	0.755	0.939
Perceived usefulness (UFN)	UFN1 UFN2 UFN3 UFN4 UFN5	0.795 0.810 0.873 0.902 0.751	0.686	0.916
Perceived susceptibility (SUS)	SUS1 SUS2 SUS3 SUS4	0.782 0.794 0.878 0.859	0.688	0.898
Perceived severity (SEV)	SEV1 SEV2 SEV3 SEV4	0.822 0.898 0.898 0.700	0.695	0.900
Self-efficacy (SEL)	SEL1 SEL2 SEL3 SEL4 SEL5	0.789 0.830 0.785 0.836 0.811	0.657	0.905
Cues to action (CUE)	CUE1 CUE2 CUE3 CUE4	0.808 0.789 0.844 0.777	0.648	0.880
Perceived value (VAL)	VAL1 VAL2 VAL3 VAL4	0.755 0.859 0.884 0.836	0.697	0.902
Perceived trust (TRU)	TRU1 TRU2 TRU3 TRU4	0.822 0.802 0.793 0.871	0.677	0.893
Consumer acceptance of ADRs (ACC)	ACC1 ACC2 ACC3 ACC4	0.866 0.869 0.882 0.844	0.749	0.923

Model fit indices: χ^2^/df = 2.106, (p < 0.05); CFI = 0.96; TLI = 0.97; RMSEA = 0.058; SRMR = 0.062.

λ denotes factor loading. AVE denotes the average variance extracted. CR denotes composite reliability.

Table 4 also shows that the measurement items were reliable. This is because all λ values were at least 0.70, and all composite reliability (CR) values were above 0.80, as proposed by Hair et al. (2010). This indicates that the measurement items measured their represented constructs reliably with internal consistency.

Additionally, Table 5 shows that the measurement items were valid on the basis of both convergent and discriminant validity. For instance, convergent validity was present, as all average variance extracted (AVE) values were larger than the value of 0.50 recommended by Kline (2015). Furthermore, discriminant validity was also present, as the squared correlation of each construct with other constructs was smaller than its corresponding AVE (Fornell and Larcker, 1981). Overall, the CFA results suggested that the theoretical model fit was good and the corresponding measurement items were reliable and valid.

**TABLE 5:** Tests for convergent and discriminant validity.

	EOU	UFN	SUS	SEV	SEL	CUE	VAL	TRU	ACC
EOU	0.755*^x^*	0.476*^z^*	0.009	0.001	0.504	0.441	0.531	0.504	0.469
UFN	0.690*^y^*	0.686	0.088	0.041	0.504	0.555	0.539	0.542	0.563
SUS	0.097	0.296	0.688	0.566	0.044	0.062	0.067	0.046	0.082
SEV	0.031	0.203	0.752	0.695	0.007	0.024	0.018	0.005	0.016
SEL	0.710	0.710	0.209	0.084	0.657	0.452	0.189	0.202	0.203
CUE	0.664	0.745	0.249	0.156	0.672	0.648	0.521	0.100	0.086
VAL	0.729	0.734	0.258	0.134	0.435	0.722	0.697	0.513	0.511
TRU	0.710	0.736	0.214	0.073	0.449	0.316	0.716	0.677	0.534
ACC	0.685	0.750	0.287	0.125	0.450	0.294	0.715	0.731	0.749

^*x*^Principal diagonal—AVEs.

^*y*^Below principal diagonal—correlations between two constructs.

^*z*^Above principal diagonal—squared correlations between two constructs.

### Analysis of structural model

**FIGURE 2:**
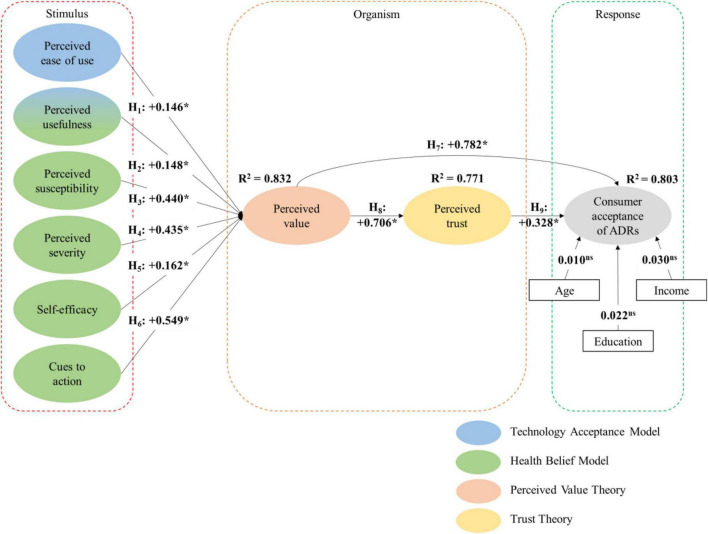
Structural model and its parameter approximations. *Indicates a significant path approximation (*p* < 0.05); ^ns^ indicates not significant; Model fit indices: χ^2^*/df* = 2.23 (*p* < 0.05); CFI = 0.95; TLI = 0.95; RMSEA = 0.06; SRMR = 0.07.

Figure 2 depicts the structural model and its approximated parameters, and control variables such as age, education and income were included in the model. Accordingly, the standardized regression estimates of the control variables are 0.010, 0.022, and 0.030, respectively.

Generally, the model fit indices presented in Figure 2 suggest good structural model fit. Additionally, the exogenous variables have strong explanatory power, as the squared multiple correlations (*R*^2^) of the endogenous variables are greater than 0.5 (Cohen, 2013).

Among the controlling variables, all have no significant effect on consumer acceptance of ADRs (*p* > 0.05). Age has no significant effect on consumer acceptance of ADRs, and this is unexpected because seniors are generally less technologically inclined, which may cause them to be less receptive to the use of ADRs. However, this may be true because COVID-19 has impacted the human population across ages and the Singapore government has been encouraging the seniors to improve their digital literacy. Similarly, education has no significant effect on consumer acceptance of ADRs, and this is intriguing as well because educated individuals tend to possess more knowledge and appreciate the benefits of using ADRs more. Finally, income also has no significant effect on consumer acceptance of ADRs. This is also surprising, as individuals with higher incomes tend to have more disposable income and greater purchasing power, enabling them to make more purchases and use ADRs more frequently as their last-mile delivery method. Nevertheless, since the control variables do not have significant effects on consumer acceptance of ADRs, it implies that there are stronger theoretical predictors of consumer acceptance of ADRs considered in the model.

All six of the technological and health belief factors, namely, perceived EOU, perceived UFN, perceived SUS, perceived SEV, SEL and CUE, have significant and positive effects on consumers’ perceptions of the value of ADRs. The respective standardized effects (β) are 0.146, 0.148, 0.440, 0.435, 0.162, and 0.549. Thus, H_1_–H_6_ are accepted. These six factors explain 83% of the variance in perceived VAL (*R*^2^ = 0.832) along with the control variables. Generally, the results are consistent with the argument of this study that both technological and health belief factors increase perceptions of the value of ADRs by contributing to functional, economic, social and hedonic utility.

For instance, when ADRs are simple and less complex to learn, use and interact with, high levels of perceived EOU will be experienced by consumers, which consequently creates economic utility from the time and resource savings compared with other delivery methods. Similarly, when ADRs are capable of conducting contactless delivery within a relatively short delivery time window and satisfying consumers’ potential desire to use the latest innovation, perceived UFN will be enhanced, which increases functional, social and hedonic utility. Apart from the technological factors, health belief factors can also contribute to the increase in the perceived VAL of ADRs. For example, when there is a high level of perceived SUS to contracting COVID-19, functional and economic utility can be created when ADRs reduce the risk of contracting COVID-19 and the loss of income by conducting contactless delivery. Simultaneously, with high levels of perceived SEV after contracting COVID-19, the economic and hedonic utility can be created with the use of ADRs, as the repercussions experienced after contracting COVID-19, such as loss of income from work absenteeism and mental health issues from prolonged isolation and stress, can be minimized. Additionally, when consumers perceive that they have the resources, knowledge and confidence to learn, use and recommend ADRs to their family and friends, this high level of SEL increases the functional and social utility. Finally, when consumers encounter positive internal and external CUE about ADRs, such as positive personal experiences, recommendations and awareness programs, consumers are more likely to choose ADRs as their preferred last-mile delivery method. Instead of merely enhancing functional utility, the hedonic utility will also be enhanced by satisfying the desire to use an innovation.

Figure 2 also reveals that consumer acceptance of ADRs is directly, positively and significantly influenced by perceived VAL (β = 0.782, *p* < 0.05). Thus, H_7_ is accepted. This is in tandem with the perceived VAL theory, which posits that if the use of ADRs offers superior value compared to other last-mile delivery methods, consumers are prone to accept and commit to the use of ADRs to experience the benefits and utilities in reality.

Additionally, consumer acceptance of ADRs is also indirectly, positively and significantly influenced by perceived VAL via perceived TRU. In particular, perceived TRU is positively and significantly influenced by perceived VAL (β = 0.706, *p* < 0.05). Therefore, H_8_ is accepted. This finding corroborates the trust theory whereby trust can be enhanced with an increase in the expected benefits and a decrease in the associated costs. For instance, an increase in perceived VAL through economic utility when consumers enjoy time and resource savings from learning and using ADRs can increase the expected benefits. Furthermore, an increase in perceived VAL by reducing the risks of contracting COVID-19 with contactless delivery can also help decrease associated costs. Both the increase in the expected benefits and the decrease in the associated costs create incentives for consumers to trust ADRs. Therefore, the above explanations support the positive and significant link between perceived VAL and perceived TRU.

Moreover, in turn, consumer acceptance of ADRs is also positively and significantly influenced by perceived TRU (β = 0.328, *p* < 0.05). Thus, H_9_ is accepted. Trust is important for consumers to accept and adopt the use of ADRs, and it can be developed when ADRs exhibit delivery expertise, reliability, integrity and competence. For example, when ADRs can conduct autonomous deliveries accurately and promptly without any human intervention, even in severe weather conditions, trust toward ADRs is developed. Consequently, this can enhance consumers’ confidence in ADRs, resulting in their acceptance. Hence, the above explanations support the positive and significant link between perceived TRU and consumer acceptance of ADRs. Combined, perceived VAL and perceived TRU explain 80% of the variance in consumer acceptance of ADRs (*R*^2^ = 0.803).

### Analysis of direct, indirect and total effects

The significance tests of a bootstrapping analysis supported the mediation effects whereby perceived VAL fully mediates the effects of the six technological and health belief variables on consumer acceptance of ADRs. Furthermore, perceived TRU partially mediates the effects of perceived VAL on consumer acceptance of ADRs. Table 6 summarizes the direct, indirect and total effects of exogenous variables on endogenous variables.

**TABLE 6:** Effects of exogenous variables on endogenous variables.

Exogenous (*i*)	Endogenous (*j*)		
	Perceived value (1)	Perceived trust (2)	Consumer acceptance of ADRs (3)
Direct effects (*a*_*ij*_) of …			
Perceived ease of use (1)	0.146	–	–
Perceived usefulness (2)	0.148	–	–
Perceived susceptibility (3)	0.440	–	–
Perceived severity (4)	0.435	–	–
Self-efficacy (5)	0.162	–	–
Cues to action (6)	0.549	–	–
Perceived value (7)	–	0.706	0.782
Perceived trust (8)	–	–	0.328
Indirect effects (*b*_*ij*_) of…			
Perceived ease of use (1)	–	0.103	0.148
Perceived usefulness (2)	–	0.104	0.150
Perceived susceptibility (3)	–	0.311	0.446
Perceived severity (4)	–	0.307	0.441
Self-efficacy (5)	–	0.114	0.164
Cues to action (6)	–	0.388	0.556
Perceived value (7)	–	–	0.232
Perceived trust (8)	–	–	–
Total effects (*c*_*ij*_) of…			
Perceived ease of use (1)	0.146	0.103	0.148
Perceived usefulness (2)	0.148	0.104	0.150
Perceived susceptibility (3)	0.440	0.311	0.446
Perceived severity (4)	0.435	0.307	0.441
Self-efficacy (5)	0.162	0.114	0.164
Cues to action (6)	0.549	0.388	0.556
Perceived value (7)	–	0.706	1.014
Perceived trust (8)	–	–	0.328

Regarding direct effects, CUE have the strongest influence on perceived VAL (*a*_61_ = 0.549), followed by perceived SUS (*a*_31_ = 0.440), perceived SEV (*a*_41_ = 0.435), SEL (*a*_51_ = 0.162), perceived UFN (*a*_21_ = 0.148) and perceived EOU (*a*_11_ = 0.146). Perceived VAL is the sole determinant of perceived TRU (*a*_72_ = 0.706). Finally, perceived VAL has a greater direct effect on consumer acceptance of ADRs (*a*_73_ = 0.782) than perceived TRU (*a*_83_ = 0.328).

Referring to the indirect effects, CUE have the most significant effect on consumer acceptance of ADRs (b*_63_* = 0.556), followed by perceived SUS (b*_33_* = 0.446), perceived SEV (b*_43_* = 0.441), perceived VAL (b*_73_* = 0.232), SEL (b*_53_* = 0.164), perceived UFN (b*_23_* = 0.150) and perceived EOU (b*_13_* = 0.148). As presented in Figure 2, the effects of the six technological and health belief factors on consumer acceptance of ADRs are fully mediated via perceived VAL and partially mediated via perceived TRU.

Finally, regarding the total effects, perceived VAL has the greatest total effect on consumer acceptance of ADRs (c*_73_* = 1.014). This is followed by CUE (c*_63_* = 0.556), perceived SUS (c*_33_* = 0.446), perceived SEV (c*_43_* = 0.441), perceived TRU (c*_83_* = 0.328), SEL (c*_53_* = 0.164), perceived UFN (c*_23_* = 0.150) and perceived EOU (c*_13_* = 0.148).

## Conclusion

### Theoretical contributions

Given that the use of ADRs is a relatively new phenomenon for last-mile delivery, there is a need to better understand consumers’ acceptance of ADRs, especially during a pandemic. Consequently, this study investigated the determinants of consumers’ acceptance of ADRs anchoring on the SOR framework and made several noteworthy contributions to the academic literature.

#### Fills the research gap

As the use of ADRs is still a relatively new phenomenon and academic research on its acceptance by consumers has been limited, this study enhances the existing literature and fills the research gap by integrating four behavioral theories to explain the determinants that influence consumers’ acceptance of ADRs. The theories used in this study, namely, the technology acceptance model, health belief model, perceived VAL theory and trust theory, are anchored on differing perspectives such as innovation acceptance, psychology and consumer utility. As a result, this forms a multi-dimensional model and provides a novel and holistic approach to examine consumers’ acceptance of ADRs during the unprecedented COVID-19 pandemic.

#### Enriches the perceived value theory

This study also supplements the perceived VAL theory by employing the technology acceptance and health belief models to analyze consumers’ acceptance of ADRs. Unlike other technology and health-related studies, which link technological and health belief factors directly to behavioral intention and consumer acceptance, this study introduces the perceived VAL theory as a mediator, which involves evaluating consumer utility from the four value dimensions. Thus, the mediating role of perceived VAL better reflects consumers’ internal cognitive and affective reactions, ultimately leading to their acceptance. Overall, the determinants explain about 80% of the variance in consumer acceptance of ADRs, which is substantial in behavioral studies (Cohen et al., 2013).

#### Offers insight into the nomological relationships

This study provides insights into the nomological relationships between the determinants influencing consumers’ acceptance of ADRs. Consistent with this study’s key arguments, perceived EOU, UFN, SUS, SEV, SEL and CUE are used by consumers to enhance perceived VAL. On the other hand, perceived VAL and perceived TRU directly influence consumer acceptance of ADRs. This finding is in line with other studies on consumer behavior and motivation, which explain that consumers’ acceptance of a product and/or service is predominantly based on the evaluation of the associated attributes (Hartono and Raharjo, 2015; Yuen and Thai, 2017). As a result, perceived VAL directly and indirectly leads to consumer acceptance of ADRs via perceived TRU.

### Strategic and policy implications

This study offers suggestions and recommendations for policymakers and ADR developers to efficiently allocate scarce resources while attempting to enhance consumer acceptance of ADRs. The implications for each determinant are discussed in order of descending importance.

The total effect analysis conducted on consumer acceptance of ADRs revealed that perceived VAL is the most important factor leading to consumers’ acceptance of ADRs. Thus, resources should first be allocated to create positive perceived VAL by improving the four value dimensions. Accordingly, policymakers can consider subsidizing the price of using ADRs, keeping it affordable for consumers (i.e., economic utility) and emphasizing other social and environmental benefits that ADRs can bring (i.e., social utility). Furthermore, ADR developers can continuously improve the quality and performance of the ADRs to meet the changing needs of consumers (i.e., functional utility), and they can also enhance the aesthetics and include interesting features to satisfy the desire of consumers to try something new while using ADRs (i.e., hedonic utility).

The next important factor is CUE. As these include both internal and external cues that facilitate the intention to use ADRs, policymakers must focus on both dimensions. Hence, internal cues can be created by conducting more ADR trials to increase consumers’ exposure to this relatively new delivery method and develop opportunities for consumers to create positive personal experiences with ADRs. Additionally, external cues like prompts on both social and traditional media can also be used to encourage and support ADRs, and existing ADR users may be incentivized when they recommend them to their family and friends through word-of-mouth.

Following that, the next two important factors are perceived SUS and perceived SEV, respectively. Since the adverse consequences (i.e., perceived SEV) after contracting COVID-19 cannot be readily influenced or controlled by human beings, it may be more feasible for policymakers to highlight to consumers that using ADRs, which have the ability to conduct contactless delivery, can help reduce the likelihood of contracting COVID-19 (i.e., perceived SUS). Contactless delivery reduces the need for human-to-human interactions, minimizing consumers’ exposure to the virus. In other words, policymakers should emphasize ADRs’ ability to meet emerging and changing societal needs during the pandemic to consumers.

Next, policymakers and ADR developers should focus on improving consumers’ perceived TRU toward ADRs. As mentioned previously, perceived TRU can be enhanced if ADRs possess the following characteristics: expertise, reliability, integrity and competence. Therefore, ADR developers should develop ADRs that can perform delivery tasks accurately and correctly without human intervention (i.e., expertise), even in severe weather conditions like heavy rainfall and poor visibility (i.e., reliability). Further, ADR developers should also ensure that security features are in place to prevent theft and pilferage during the delivery process (i.e., integrity). Finally, more ADR trials can be conducted to ensure that ADR delivery is as effective as other conventional human-intervened delivery methods (e.g., home delivery), and policymakers may use positive findings from such trials to market the use of ADRs to consumers (i.e., competence).

Following perceived TRU is SEL. As SEL relates to consumers’ confidence in facilitating decisions and allocating resources in learning and using ADRs, policymakers can consider conducting demonstrations and training on using ADRs for consumers to improve their confidence in learning and using ADRs. The use of ADRs can also be marketed to consumers who feel that using them is consistent with their purchase or delivery needs.

Therefore, policymakers and ADR developers should allocate resources to enhancing the perceived UFN of ADRs. This can be done by promoting the various benefits of using ADRs over other delivery methods (e.g., self-collection from parcel lockers) to consumers and continuously improving ADRs according to the feedback gathered from consumers while attempting to meet the consumers’ needs better.

Finally, policymakers should also conduct infrastructural developments and improvements in Singapore, such as enabling 5G networks and creating more barrier-free access, to allow for the smooth operation of ADRs. This can positively influence consumers’ perceptions of the EOU of ADRs.

### Limitations and recommendations

Various limitations exist despite the contributions made by this study. This study was conducted in Singapore, a densely populated island city-state with unique demographic characteristics. With the stringent safe management measures in place to stabilize the COVID-19 situation and protect the overall healthcare system in Singapore, consumers’ perceptions of ADRs and COVID-19 may be influenced. Thus, the results should be interpreted with caution, as they may not be applicable to other less-populated or less-developed geographical regions and countries. Therefore, further study can be conducted to cross-validate with other geographical contexts and examine the generalizability of the theoretical model.

In addition, as this study was conducted at a particular time during the pandemic, the level of influence of each determinant may not be reflected across the different pandemic stages. Therefore, a longitudinal observation and assessment should be carried out. Accordingly, future research and surveys should be conducted over a prolonged period to examine the diffusion of consumers’ ADR acceptance.

## Data availability statement

The raw data supporting the conclusions of this article will be made available by the authors, without undue reservation.

## Ethics statement

The studies involving human participants were reviewed and approved by the NTU-IRB. The patients/participants provided their written informed consent to participate in this study.

## Author contributions

KY: conceptualization, survey design, data collection, and writing and revision. LC: revision and editing. YL: data collection and writing. XW: writing, editing, and revision. All authors have read and agreed to the published version of the manuscript.
